# Microfluidic EDGE emulsification: the importance of interface interactions on droplet formation and pressure stability

**DOI:** 10.1038/srep26407

**Published:** 2016-05-27

**Authors:** Sami Sahin, Olesya Bliznyuk, Ana Rovalino Cordova, Karin Schroën

**Affiliations:** 1 Wageningen University, Food Process Engineering, Bornse Weilanden 9, 6708 WG Wageningen, The Netherlands; 2 Wageningen University, Food Quality Design, Bornse Weilanden 9, 6708 WG Wageningen, The Netherlands

## Abstract

The fact that interactions of components with interfaces can influence processes is well-known; e.g. deposit accumulation on heat exchangers and membrane fouling lead to additional resistances against heat and mass transfer, respectively. In microfluidic emulsification, the situation is even more complex. Component accumulation at the liquid/liquid interface is necessary for emulsion stability, while undesired at the solid/liquid interface where it may change wettability. For successful emulsification both aspects need to be controlled, and that is investigated in this paper for o/w emulsification with microfluidic EDGE devices. These devices were characterised previously, and can be used to detect small wettability changes through e.g. the pressure stability of the device. We used various oil/emulsifier combinations (alkanes, vegetable oil, surfactants and proteins) and related droplet size and operational pressure stability to component interactions with the solid surface and liquid interface. Surfactants with a strong interaction with glass always favour emulsification, while surfactants that have week interactions with the surface can be replaced by vegetable oil that interacts strongly with glass, resulting in loss of emulsification. Our findings clearly show that an appropriate combination of construction material and emulsion components is needed to achieve successful emulsification in microfluidic EDGE devices.

Emulsions, which are mixtures of oil and water, are conventionally prepared using high pressure homogenizers and colloid mills. These devices apply high shear to break-up initially large emulsion droplets into smaller ones^1^ that subsequently need to be stabilised by emulsifiers. The time scale for droplet break-up is very short, often leading to insufficient stabilisation and recoalescence^2^. To remediate this, repeated homogenization in combination with excess emulsifier is used, which are both undesirable from an economic point of view. Typically 95% of the energy input may be lost as heat, which also can lead to degradation of heat-sensitive ingredients. Besides, the droplet size distributions are rather wide, with a typical coefficient of variation (CV) of 40%, which influences the physical stability of the emulsions in a negative way^3^.

In the last two decades, direct emulsification systems (i.e. membranes and microfluidic devices) have been introduced for producing monodisperse emulsions at low energy consumption^1^,^4^,^5^. Unlike in homogenization, in these systems droplets are made at their final size without further refinement. Microfluidic emulsification devices can be divided into two categories based on the droplet formation mechanism: shear-based^6^,^7^,^8^ and spontaneous or interfacial tension driven^9^,^10^,^11^. In shear-based systems (e.g. T-, and Y-junctions) the flow of both phases influences the droplet size, while in spontaneous systems (e.g. microchannels and EDGE devices) only the dispersed phase does so. For an overview of microfluidic emulsification systems, we refer the interested reader to recent reviews^1^,^4^,^5^,^12^.

For droplet formation in spontaneous emulsification devices, the surfaces of the droplet formation units should be wetted by the continuous phase, and the wettability should remain unchanged to maintain successful operation. For o/w emulsification with silicon microchannels, Kobayashi *et al.*^13^ showed that monodisperse emulsions could be prepared successfully using anionic and non-ionic surfactants, while cationic surfactants resulted in either polydisperse emulsions (i.e. non-uniform wettability) or continuous outflow of the dispersed phase (i.e. wetting by the dispersed phase). Saito *et al.*^14^ could prepare monodisperse emulsions with straight-through microchannels when using bovine serum albumin (BSA), β-lactoglobulin (β-lac), soybean flour, and whey protein, while this was not possible with γ-globulin, lysozyme and egg white protein. Saito and co-workers related the differences in observed emulsification behaviour to net electrical charges of the proteins, which is in line with the explanation of Kobayashi *et al.*^13^ for the low molecular weight surfactants. Van Dijke *et al.*^2^ reported successful preparation of emulsions using whey protein concentrate and skim milk in combination with various oils in EDGE devices. These reports clearly point to the importance of component interactions on droplet formation behaviour in microfluidic devices, however, this has not been investigated systematically so far and therefore the explanations are not conclusive.

In this paper, we chart emulsification behaviour in microfluidic EDGE devices, and link this to component interactions with solid surfaces and liquid interfaces. To allow this, the solid surfaces needed to be rigorously cleaned as described in the materials and methods section. Various emulsifiers and oils were tested, and from the observations, guidelines for successful operation could be deduced.

## Results and Discussion

### Emulsification behaviour

**Figure 1:**
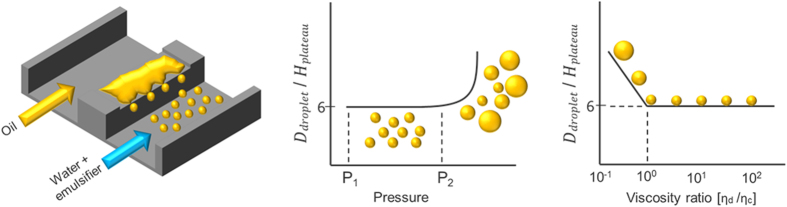
Droplet formation characteristics in EDGE microchips. Monodisperse droplets form between the break-through (P_1_) and blow-up pressures (P_2_), and beyond the blow-up pressure droplets become polydisperse. Between P_1_ and P_2_, the droplet diameter scales with the plateau height by a factor of 6 at high viscosity ratios (>1), and at low viscosity ratios droplet size increases with decreasing ratio.

Before discussing the results, the characteristics of EDGE devices are summarized to facilitate interpretation of the results by our readers. Figure 1 is a schematic impression of droplet formation in EDGE chips. The oil is pushed onto the shallow plateau over which an oil film flows toward the deeper continuous phase channel. Upon reaching the plateau edge, the oil film is spontaneously transformed into many spherical droplets along the entire plateau. The formed droplets are carried to the channel exit by the continuous phase flow; please note that droplets snap off spontaneously by interfacial tension forces, not through shear forces exerted by the flow of continuous phase. The simultaneous formation of multiple droplets is also the greatest difference with other microfluidic emulsification devices in which one droplet is formed per droplet formation unit at a time. As depicted in Fig. 1, monodisperse droplets start forming at break-through pressure (P_1_), and they become polydisperse at blow-up pressure (P_2_). Between these pressures, the diameter of the droplets is ~6 times the height of the plateau. This scaling is valid at a viscosity ratio >1 (dispersed/continuous), and at lower ratios the scaling factor increases with decreasing viscosity ratio^15^,^16^. In the current study, the viscosity ratios are 3.47 and 50 for hexadecane and sunflower oil, respectively.

The minimum pressure required for oil to invade the plateaus is determined by the Young-Laplace equation^2^:


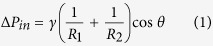


where 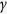 is the interfacial tension, *R*_1_ and *R*_2_ are the radii of the oil-water interface, and *θ* is the contact angle. *R*_1_ and *R*_2_ are defined by the height (2 μm) and width of the plateau (500 μm), and because the latter is much greater than *R*_*1*_, its effect on equation 1 can be neglected. The pressure at which droplet formation starts is determined by 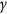/*R*_*1*_, as is the case in classic maximum bubble pressure tests that are used to determine interfacial tension^17^. Under the conditions used for EDGE emulsification, the interfacial tension is expected to be equal to its equilibrium value. In our experiments, the minimum pressure at which droplet formation started was mostly slightly higher than the invasion pressure (few mbar for 2 μm height plateaus), indicating good wetting of the surface.

### Low molecular weight surfactants

**Figure 2:**
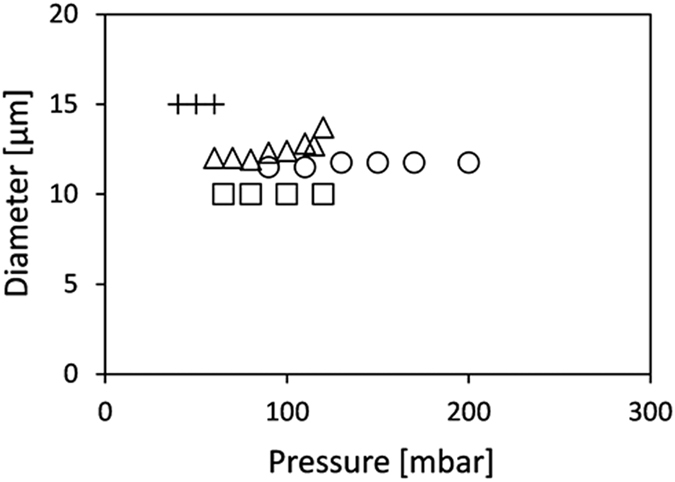
Droplet size as function of applied pressure: hexadecane/SDS (∆), hexadecane/Tween 20 (○), sunflower oil/SDS (+), sunflower oil/Tween 20 (◻). The CVs are below 10%, with the exception of sunflower oil/SDS for which the presented data have a CV < 20%.

Figure 2 shows the diameter of hexadecane and sunflower oil droplets stabilised by Tween 20 and SDS as a function of applied pressure. With Tween 20, monodisperse hexadecane and sunflower oil droplets were obtained, albeit at a narrower pressure range for sunflower oil. With SDS, monodisperse hexadecane droplets could be produced successfully, while sunflower oil droplet formation was only possible for a very narrow pressure range (40–60 mbar), and the droplets were larger than expected (15 compared to 12 μm) and more polydisperse, making sunflower oil/SDS an unsuccessful combination.

As mentioned before, droplet formation units need to be wetted by the continuous phase for successful emulsification. Tween 20 can adsorb tightly onto glass and cannot be displaced by oil, making the pre-adsorbed surfactant layers the real contact surface that favoured stable droplet formation for both oils (Fig. 2). Because SDS does not form tightly bound films on the glass surface, monodisperse droplets could only be made with hexadecane that does not interact strongly with the hydrophilic glass surface. Triglycerides, the main constituents of sunflower oil, can form tightly bound films on the glass, and when given sufficient time sunflower oil was able to replace SDS from the surface, thereby hindering monodisperse droplet formation. This was even more evident when sunflower oil was introduced onto the plateau before it was exposed to the SDS solution; monodisperse droplet formation was not possible at any pressure. Formation of an oil film in time may also explain contradicting results from literature, which report success and failure for seemingly the same (vegetable) oil/emulsifier combinations^18^.

**Figure 3:**
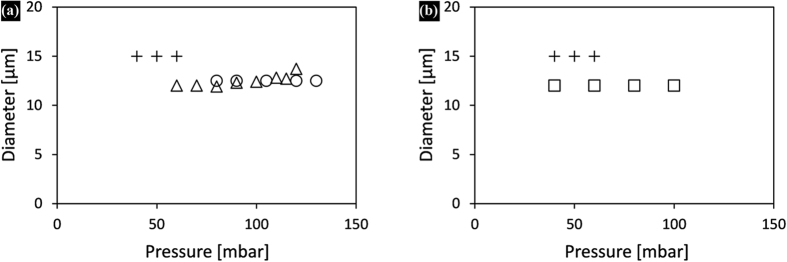
(**a**) Droplet size as a function of applied pressure for SDS stabilised emulsions prepared with sunflower oil (+), hexadecane (Δ), paraffin-hexadecane mixture (○), (**b**) comparison of sunflower oil/SDS droplet formation when using untreated (+) and WPI pre-treated (◻) glass chips. The CVs for the presented data points are below 10%, except for (+) which is considered unsuccessful.

In order to exclude a possible effect of the high viscosity of sunflower oil on the observed behaviour, an oil with the same viscosity was used (paraffin-hexadecane mixture), and the obtained droplet size and pressure stability were comparable to that found for hexadecane (Fig. 3a). This clearly suggests that the large (polydisperse) sunflower oil droplets obtained with SDS were caused by the interactions of sunflower oil with the glass surface. Further, when allowing whey protein to adsorb onto the plateau and thus form an irreversibly deposited hydrophilic layer that cannot be replaced by oil, sunflower oil/SDS could generate monodisperse droplets at the expected droplet size and appreciable pressure stability (Fig. 3b). These results clearly indicate that component interactions with the solid surface are of great relevance for successful emulsification.

### Proteins as emulsifiers

**Figure 4:**
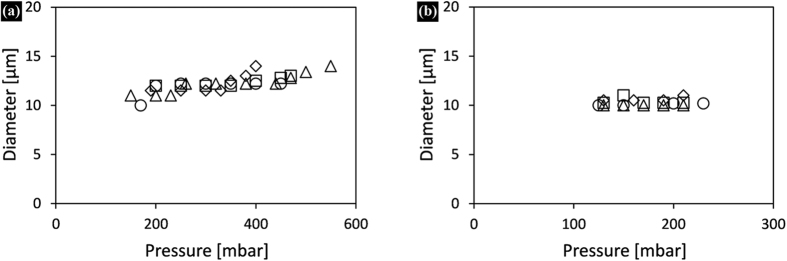
(**a**) Average diameters of (a) hexadecane and (b) sunflower oil droplets as a function of applied pressure when using 5% solutions of WPI (○), α-lac (Δ), β-lac (◽), BSA (◇) as continuous phase. The CVs for the presented data points are below 10%.

In Fig. 4 the average sizes of hexadecane (Fig. 4a) and sunflower oil (Fig. 4b) droplets prepared with various proteins were plotted as a function of applied pressure. Monodisperse droplets of both oils were successfully produced when using 5% solutions of WPI, α-lac, β-lac and BSA at neutral pH. Stable droplet formation was maintained for several hours, clearly indicating that all proteins formed a hydrophilic film on the solid surface, and more importantly they could not be displaced by the oil during emulsification. As with the surfactants, 12 micrometer hexadecane and ~10 micrometer sunflower oil droplets were produced with proteins.

The droplet size in EDGE emulsification is not dependent on the interfacial tension and contact angle but the pressure at which droplet formation starts is influenced by these factors (see equation 1). For sunflower oil emulsions all proteins performed similarly (Fig. 4b), while some small differences were noticed for hexadecane emulsions (Fig. 4a). Very similar droplet sizes and pressure ranges were obtained with β-lac and WPI, which is logical as β-lac is the main constituent of WPI. With α-lac, and BSA slightly different droplet sizes and pressure ranges were obtained, as will be discussed in more detail later.

**Figure 5:**
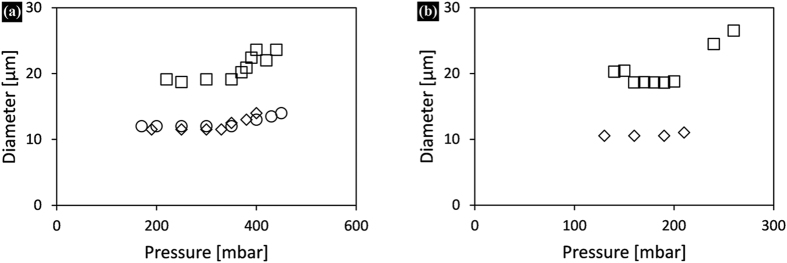
Average diameters of (**a**) hexadecane and (**b**) sunflower oil droplets as a function of applied pressure when using 5% BSA solutions at pH 3 (○), 4.8 (◽) and 7 (♢). The CVs for the presented data points are below 10%.

Depending on the pH, adsorbed proteins are tightly or loosely bound to the surfaces, and also interactions between adsorbed molecules can change drastically. We investigated this through emulsification of hexadecane and sunflower oil with BSA that is the most used protein in adsorption studies. We used 5% BSA solutions at pH 3, 4.8 (isoelectric point) and 7; the average droplet sizes obtained as a function of applied pressure are shown in Fig. 5.

At pH 3 monodisperse hexadecane droplets were prepared at similar pressure stabilities and sizes as obtained at pH 7 (Fig. 5a); however, it was not possible to prepare monodisperse sunflower oil droplets (not shown in the figure). At this pH the interaction between protein and surface is weak, and the sunflower oil has strong interaction with the surface (leading to irregular droplet formation), contrary to hexadecane that does not have strong interactions as shown previously (hexadecane/SDS; Fig. 3a). At the isoelectric point at which BSA is a poor emulsifier, much larger (19 μm) but surprisingly very monodisperse droplets were found for both oils (Fig. 5a,b). The change in sunflower oil droplet formation as function of pH is partly in line with the findings of Saito *et al.*^14^ who could prepare BSA stabilised monodisperse soybean oil droplets through microchannel emulsification at pH values above the isoelectric point, whereas they noted loss of emulsification behaviour below the isoelectric point. The different pH dependency of the two systems may be attributed to the much smaller microchannels that are expected to be more sensitive to wettability changes than EDGE devices.

### Pressure stability and system productivity

As illustrated in the previous figures, the pressure range in which monodisperse droplet formation takes place depends strongly on the components used. The pressure stability is not only important for operational stability but also for the productivity of the system; the wider the pressure stability, the higher the droplet formation frequency. Table 1 summarizes the pressure stability obtained with various oil/emulsifier combinations and the droplet formation frequencies obtained at the maximum pressures.

As described in the introduction, the minimum pressure at which droplet formation takes place is a function of the interfacial tension. As shown in Table 1, for a given emulsifier higher pressures were needed to start droplet formation with hexadecane (~45 mN/m, bare surface) compared to sunflower oil (~25 mN/m, bare surface). Similarly, droplet formation started at lower pressures with surfactants that are known to lower interfacial tension more than proteins at the concentrations used here.

Regarding the pressure stability, proteins outperform surfactants, which is even more noticeable for hexadecane. In line with the general rule that higher pressure stability implies higher droplet formation frequency, for all protein stabilized hexadecane droplets (except BSA at pH 7) droplets were generated at much higher frequencies than with any of the surfactant systems (Table 1). Although there is a link between pressure stability and droplet formation frequency, differences between various proteins cannot be explained solely based on that. For example, α-lac at 440 mbar yielded more than twice as many droplets as β-lac at 470 mbar. Similarly, the droplet formation frequency obtained with BSA at 400 mbar was comparable to those obtained with SDS and Tween 20 at much lower pressures (<200 mbar). Therefore, we can conclude that the droplet formation frequency is also related to the structure and adsorption behaviour of the proteins at the solid/liquid and liquid/liquid interfaces. This inference is supported by the significantly higher productivity of hexadecane droplets using BSA at pH 3 compared to pH 7. Also Maan *et al.*^19^ noted changes in pressure stability in EDGE microchips for hexadecane/Tween emulsions when varying the surface properties. For instance, they observed a wider pressure stability when using microchips with aldehyde terminated SAMs (more hydrophilic), and they did not observe stable droplet formation with phenyl terminated SAMs (more hydrophobic). The changes were related to the adsorption behaviour of Tween on different SAMs.

Compared to hexadecane, sunflower oil droplets formed at much lower rates, and this was a result of its much higher viscosity (by a factor of 15) and lower pressure ranges. Similar frequencies were obtained with all proteins, the only exception being BSA that gave the lowest frequency also for hexadecane. With Tween 20, the frequencies were similar as found with proteins, and SDS could not be used with sunflower oil as stated in earlier sections.

In summary, Table 1 clearly illustrates the differences in pressure stabilities and droplet formation frequencies, for which we hold both the interaction of the surfactant or protein with the solid/liquid and liquid/liquid interface responsible. Besides, the interaction of the oil with the solid/liquid interface is of great relevance for stable operation; strong interaction of the oil with the solid surface leads to irregular droplet formation, or even failure to make emulsions. For this reason, when emulsifying an oil that has strong interaction with the surface, the emulsifier should be chosen such that it cannot be replaced from the surface by the oil. In this respect, permanent surface modification is of great relevance^19^,^20^,^21^,^22^, but also here any adsorbed components should be taken into account.

## Conclusions

We have shown that interactions between the oil, emulsifier and surface are decisive for the droplet formation behaviour in microfluidic EDGE devices, and we expect this also to be true for other microfluidic devices. Compared to surfactants, significantly higher pressure stabilities and productivities were achieved when proteins were used as emulsifiers. We strongly believe that our findings provide new leads for successful emulsification using microfluidic devices and for their upscaling, which requires consideration of construction material, product formulations, and throughput as a ‘total package’, not as individual aspects, as is the common practice nowadays.

## Experimental

### Chemicals

For the preparation of o/w emulsions, hexadecane (C_16_H_34_, ReagentPlus^®^, 99%, Sigma-Aldrich), viscous paraffin (MERCK) and sunflower oil purchased from a local supermarket were used as dispersed phase. Sunflower oil was filtered through 0.45 μm filters before use. As continuous phase, 0.5% w/w sodium dodecyl sulphate (SDS) and 2% w/w Tween 20 solutions, and 5% w/w solutions of several proteins were used: whey protein isolate (>90%, BiPRO, Davisco), which consists of 72% β-lactoglobulin, 24% α-lactalbumin and 4% BSA; calcium-depleted α-lactalbumin (>95%, Davisco); β-lactoglobulin (>95%, obtained by selective precipitation of commercial WPI); and bovine serum albumin (95%, Sigma). All aqueous solutions were prepared using Ultrapure MilliQ water and filtered using 0.22 μm filters. The pH of the protein solutions was ~7, and no adjustment was made unless otherwise stated.

### Design and fabrication of microchips

**Figure 6:**
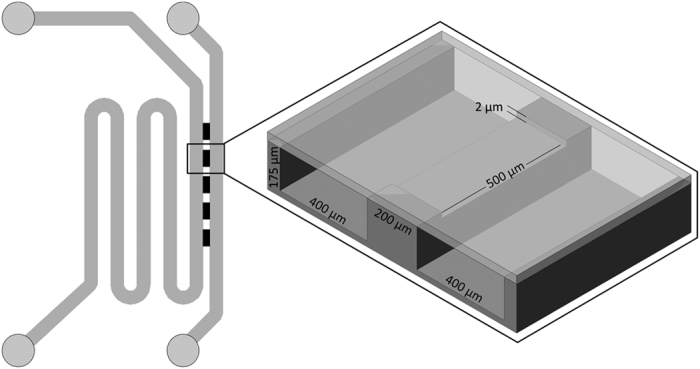
The EDGE microchips consist of feed channels for the water and oil phase (175 μm deep and 400 μm wide). These channels are bridged through five identical plateaus of 200 × 500 × 2 μm (length × width × height), each separated by 300 μm bonding space.

The microfluidic EDGE chips were made in glass using deep reactive ion etching (DRIE) technique (Micronit Microfluidics, Enschede, The Netherlands). Figure 6 depicts the layout of the microchips and the droplet formation unit with its respective dimensions; the chips consist of five identical shallow plateaus of 2 μm deep (black rectangles) bridging the dispersed and continuous phase (meandering) channels that are 175 μm deep and 400 μm wide. The shallow plateaus and deep channels were etched into two separate glass substrates which were afterwards bonded together. The closed chips were oxidized and cleaned to make them hydrophilic and suitable for o/w emulsification.

### Cleaning of microchips

Before their first use, microchips were placed in a furnace and baked at 550 °C for 2 hours. To clean the microchips after use, they were first flushed with ethanol, and subsequently sonicated in piranha solution (a mixture of H_2_SO_4_ 96% and H_2_O_2_ 33% in 3:1 v/v ratio) for an hour.

### Experimental setup and emulsification

The microchip was placed in a Fluidic Connect PRO chip holder (Micronit Microfluidics, Enschede, The Netherlands), and the channel inlets and outlets of both phases were connected to the outside world through 1/16″OD PEEK tubing with an inner diameter of 0.030″. Both phases were introduced into the chip using a microfluidic flow control system (Elveflow^®^, Paris, France). Owing to the design of the EDGE devices, the pressure drop inside the channels was ~1 mbar (negligible compared to the invasion pressure), which implies all plateaus operated at the same pressure drop. First the continuous phase (i.e. emulsifier solution) was fed into the chip for half an hour to allow emulsifier adsorption on the plateau surface. Then, the oil was pushed through the dispersed phase channel, and after a steady oil flow developed the channel outlet was blocked. The dispersed phase pressure was increased step by step to scan the window of monodisperse droplet formation.

The microchip was placed on a microscope stage, and the droplet formation process was observed via a high-speed camera (MotionPro HS-4, IDT Inc., Tallahassee, FL, USA) connected to the microscope (Axiovert 200 MAT, Carl Zeiss b.v., Sliedrecht, The Netherlands). The microscope was equipped with a set of objectives up to 100× and an optavar with factors of 1×, 1.6× and 2.5×, and the high-speed camera was able to acquire images at up to 5130 fps at the maximum resolution of 512 × 512. The combination of frame rate and magnification was limited by the amount of the light, so the best combination was sought to acquire images for post processing.

### Image analysis

At each process setting, the emulsification process was allowed to stabilise for 5–10 minutes prior to imaging. The images used for sizing were always acquired at the same magnification to eliminate measurement errors inherent to pixel distribution. The size and size distribution of the droplets were determined using image analysis software. Up to 50 droplets were analysed and averaged, which is an established procedure in our lab for monodisperse droplets. The droplet size distribution was expressed in coefficient of variation, CV, which is defined as:


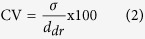


where 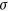 is the standard deviation and 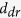 is the number-average droplet diameter. Droplet formation frequency was determined by analysing 1000 subsequent frames that were acquired at 500–5000 fps.

## Additional Information

**How to cite this article**: Sahin, S. *et al.* Microfluidic EDGE emulsification: the importance of interface interactions on droplet formation and pressure stability. *Sci. Rep.* **6**, 26407; doi: 10.1038/srep26407 (2016).

## Figures and Tables

**Table 1:** Maximum droplet formation frequencies obtained with different oil/emulsifier combinations.

Emulsifier	Hexadecane			Sunflower oil		
	Pressure range [mbar]	@ maximum pressure		Pressure range [mbar]	@ maximum pressure	
		Diameter [μm]	Frequency per 500μm plateau width [Hz]		Diameter [μm]	Frequency per 500 μm plateau width [Hz]
SDS	60–115	13	160	40–60	15	12
SDS/WPI^a^				40–100	12	67
Tween 20	90–200	12	200	65–120	10	50
WPI	170–450	12	1221	125–220	10	28
α-lac	150–550	14	5500	130–210	10	33
β-lac	190–470	13	1073	130–210	10	28
BSA pH 7	190–400	14	150	130–210	11	8
BSA @ pI	220–440	24	526	150–260	26	31
BSA pH 3	190–450	14	1255	Polydisperse	–	–

^a^Prior to sunflower oil/SDS experiment, the channel surfaces were modified through protein adsorption.
